# Changing patterns of the prevalence of burnout and secondary traumatic stress in health‐system pharmacists throughout the COVID‐19 pandemic

**DOI:** 10.1002/jac5.1632

**Published:** 2022-05-09

**Authors:** Rima A. Mohammad, Adam M. Jones, John S. Clark

**Affiliations:** ^1^ Department of Pharmacy University of Michigan College of Pharmacy and Michigan Medicine Ann Arbor Michigan USA; ^2^ Department of Pharmaceutical Care University of Iowa Hospitals & Clinics Iowa City Iowa USA

**Keywords:** burnout, compassion, coronavirus, COVID‐19, pharmacists, ProQOL, stress, trauma

## Abstract

**Introduction:**

The demands posed during the coronavirus disease 2019 (COVID‐19) pandemic have led to greater stress and frustration, which in turn can fuel exhaustion, cynicism, secondary traumatic stress (STS), and burnout. More evidence is needed regarding the prevalence of burnout and STS throughout the pandemic.

**Objectives:**

The aim of this study was to describe the changing pattern of the prevalence of burnout and STS in health‐system pharmacists throughout the pandemic (early to 20 months into the pandemic).

**Methods:**

A cross‐sectional, listserv‐based online survey was conducted in health‐system pharmacists. The survey was administered between April and May 2020 (early group) and again between October and December 2021 (20‐month group). The survey questionnaire included demographics, employment characteristics, COVID‐19‐related questions, survey of respondent's perceptions of prevalence and severity of burnout, and Professional Quality of Life Scale (ProQOL) which assessed compassion satisfaction and fatigue (burnout and STS).

**Results:**

A total of 1126 health‐system pharmacists completed the survey (484 in the early group and 642 in the 20‐month group). Based on respondents' self‐rating of burnout, significantly more respondents reported feeling burned out in the 20‐month group vs the early group (69% vs 47.7%; *P* < .001). Based on ProQOL, significantly more respondents were identified with moderate–high likelihood of burnout (83.8% vs 65.3%; *P* < .001) and moderate–high probability of STS (63.2% vs 51.4%; *P* < .001) in the 20‐month group vs the early group. Approximately 99% of respondents in both groups were identified with moderate–high probability of compassion satisfaction.

**Conclusion:**

Twenty months into the COVID‐19 pandemic, almost 83% of health‐system pharmacist respondents were identified with burnout, 63% with STS, and 99% with compassion satisfaction. These rates are significantly higher compared with rates early in the pandemic. Unfortunately, the development of burnout and STS in these pharmacists may lead to several work‐related consequences (eg, increase risk of medical errors); therefore, further studies are critical to develop and assess effective interventions to address the long‐term effects of the pandemic and well‐being of health‐system pharmacists.

## 1 INTRODUCTION

As the coronavirus disease 2019 (COVID‐19) pandemic continues and surges throughout the United States (US), increased work demand and effort on health care professionals, especially in health care systems, can lead to increased burnout, psychological symptoms, and secondary traumatic stress (STS).^1^, ^2^, ^3^ A recent survey conducted by the American Medical Association of health care professionals showed that 38% reported anxiety and depression, 43% suffered from work overload, and 49% had burnout (high or very high).^4^ Allied health care professionals (speech therapists, occupational therapists, and social workers) reported the highest rates of burnout compared with other health care professionals. The prevalence of burnout and STS in pharmacists during the COVID‐19 pandemic had been described in recent studies. One survey study showed that over half of health‐system pharmacists were identified with burnout, half with STS, and three fourths with compassion satisfaction during the early period of the COVID‐19 pandemic.^5^ Another survey study conducted in pharmacists (42.2% in hospital and 39.9% in community setting) during the early period of the pandemic showed that emotional exhaustion and depersonalization scores were higher during the pandemic compared with pre‐pandemic, which indicated increased burnout.^6^ One survey study in pharmacists during the early period of the pandemic showed that about half of pharmacists reported increased feelings of physical and emotional exhaustion at work, 40% reported experiencing anxiety, and 25% reported more sadness or depression.^7^ Based on these studies, the COVID‐19 pandemic has put significant pressure and stress on health care professionals, especially pharmacists.

The demands posed during these unprecedented times perhaps have led to greater stress and frustration, which in turn can fuel the exhaustion, cynicism, and inefficacy of burnout.^3^ This process has also been described as compassion fatigue, STS, and vicarious traumatization. Unfortunately, compassion fatigue, which includes burnout and STS, can lead to medical errors, impact standards of patient care and relationships with other co‐workers, and lead to physical and mental health conditions.^3^ Additionally, compassion satisfaction can also be present which occurred when a person is professionally satisfied with their position. The goal is for a person to have more compassion satisfication to increase the chance to overcome compassion fatigue related to their job.^3^ Therefore, it is crucial to evaluate the extent of compassion fatigue and satisfaction in health care professionals, especially health‐system pharmacists, throughout the COVID‐19 pandemic. Currently, there are no studies that evaluated the changing pattern of the prevalence of burnout and STS, and perception related to burnout in health‐system pharmacists throughout the COVID‐19 pandemic. This study evaluated the prevalence of burnout and STS in health‐system pharmacists early in the pandemic compared with 20 months into the pandemic.

## 2 METHODS

### 2.1 Study design

A cross‐sectional, professional pharmacy organization listserv‐based online survey was conducted with a target group of health‐system pharmacists across the US. The local institutional review board approved the study prior to initiation. The primary objective of this study was to compare the prevalence of burnout and STS in health‐system pharmacists early in the COVID‐19 pandemic (early group) to 20 months into the pandemic (20‐month group). The survey was sent out to five communities that include health‐system pharmacists through the American Society of Health‐System Pharmacists (ASHP) listservs and was completed anonymously and on a voluntary basis. These communities included (1) COVID‐19 (n = 53 000), (2) Clinician Well‐Being and Resilience (n = 391), (3) New Practitioners (n = 7500), (4) Inpatient Practitioners (n = 20 800), and (5) Pharmacy Practice Leaders (n = 14 500). A response rate calculation was not performed because targeted communities include non‐health‐system pharmacists and therefore would not represent health‐system pharmacists who responded to the survey. Pharmacists who indicated that they practice in a U.S. health‐system were included in the study. Incomplete survey responses were excluded from the study. The survey was initially sent to the members of the listservs between April 21, 2020 and May 20, 2020, which assessed the initial prevalence of burnout and STS in health‐system pharmacists early in the pandemic. The same survey was sent to the same listservs between October 15, 2021 and December 31,2021, which assessed the prevalence of burnout and STS in these pharmacists 20 months into the pandemic. The methods of the initial study have been described in a previous publication.^5^

### 2.2 Survey design and data collection

The survey questions were developed through Qualtrics Survey Software, Version 2020 (Qualtrics, Provo, UT) by investigators, reviewed by pharmacists and managers, and modified based on feedback. The survey was also tested on 10 internal health‐system pharmacists and further revised the survey based on results and feedback from these pharmacists. This established face and content validity of the survey. The survey questionnaire included 62 items assessing demographics, employment characteristics, COVID‐19‐related questions, respondents' perception of burnout prevalence, respondents' self‐rating of burnout, and the Professional Quality of Life Scale (ProQOL) questionnaire. Demographics included age, gender, ethnicity, marital status, children status, loan status, years in practice, areas of practice, place of employment, institution type and size, location of employment, employment position, full or part‐time employment, certifications, highest degree held, post‐graduate training, and other questions such as hobby, sleep, and exercise status. Employment location was further categorized into two groups: (1) Location in top 10 states with the highest rates of COVID‐19 infection, and (2) Other states outside of the top 10 states. The top 10 states for the initial study (early group) included New York, New Jersey, Illinois, Massachusetts, California, Pennsylvania, Michigan, Texas, Florida, and Maryland. The top 10 states for the follow‐up study (20‐month group) included New York, Wisconsin, Illinois, Massachusetts, California, Pennsylvania, North Carolina, Texas, Florida, and Ohio. The COVID‐19‐related questions included impact on employment hours, position, responsibilities, and salary or benefits, and impact on childcare and significant other's employment if applicable. The Physician Work Life Study (PWLS) Single item was used to assess respondents' self‐rating of burnout.^8^

The ProQOL was used to measure the negative and positive effects of helping others who experience suffering and trauma.^9^, ^10^ This tool has been used to assess both compassion satisfaction and fatigue in health care professionals in extremely stressful events. Therefore, the ProQOL was used in this study in the setting of an extremely stressful event such as the COVID‐19 pandemic. Compassion fatigue is further categorized as burnout and STS. An individual score is provided for each statement and each statement is scored based on the ProQOL categories, which includes (1) Compassion satisfaction, (2) Burnout, and (3) STS. Prior to calculating the overall score based on ProQOL category, the scores of the five items related to positive experiences were reversed (ie, if 1 is selected, changed to 5). Based on calculated ProQOL scores, a score of 22 or less indicated the low likelihood of the specific ProQOL category, a score between 23 and 41 indicated the moderate likelihood of that category, and a score of 42 or more indicated the high likelihood of that category. More details regarding the ProQOL assessment tool were described in the published initial study.^5^

### 2.3 Study outcomes

The primary outcome of the study was the prevalence of burnout and STS in health‐system pharmacists 20 months into the COVID‐19 pandemic (20‐month group) compared with early in the pandemic (early group). Descriptive statistics were used to describe respondents' characteristics, ProQOL scores and categories, burnout characteristics, and COVID‐19‐related factors. The chi square test was used to compare categorical data (characteristics, ProQOL categories, burnout characteristics, and COVID‐19‐related factors) and *t*‐test was used to compare continuous data (age) between the groups. All statistical analyses were performed using SPSS 28 (SPSS, Armonk, NY).

## 3 RESULTS

The survey was started by 1421 health‐system pharmacists and completed by 1126 (484 in the early group and 642 in the 20‐month group). Respondent and employment characteristics were similar in both the early and 20‐month groups. Survey respondents had an average age of 42 years old, and most were female (approximately 72%), Caucasian (86%), married (approximately 70%), and have children (approximately 60%). The majority of survey respondents stated they had a hobby (70%), exercised regularly (approximately 60%), regularly got 7‐9 hours of sleep (55% in the early group vs 61% in the 20‐month group), and had no student loans (63%). Almost half of survey respondents practiced in a top 10 state with the highest rates of COVID‐19 infections, half of respondents were in a management position, and three‐fourths worked in an inpatient hospital setting. Table 1 summarizes the demographics and characteristics of respondents and employment between the two groups.

**TABLE 1:** Respondents and employment characteristics

Characteristic	Early group (n = 484)	20‐Month group (n = 642)	*P* value
Age, year, mean ± SD (range)	41.95 ± 11.68 (21‐70)	42.8 ± 10.9 (24‐72)	.19
Gender, n (%)			
Female	346 (71.5)	472 (73.6)	.67
Ethnicity, n (%)			
White or Caucasian	418 (86.4)	550 (85.8)	.77
Marital status, n (%)			
Married	337 (69.6)	464 (72.4)	.31
Children, n (%)			
Yes	286 (59.1)	400 (62.4)	.26
Number of children, mean ± SD (range)	2.24 ± 0.91 (1–6)	2.21 ± 0.93 (1‐6)	.60
Age of children, n (%)			
0–9	129 (45.1)	174 (43.5)	.68
>10	157 (54.9)	226 (56.5)	
Hobby, n (%)			
Yes	342 (70.7)	446 (69.6)	.69
No	142 (29.3)	195 (30.4)	
Exercise regularly, n (%)			
Yes	296 (61.2)	374 (58.3)	.34
No	188 (38.8)	267 (41.7)	
Regularly get 7‐9 h of sleep, n (%)			
Yes	294 (60.7)	356 (55.5)	.08
No	190 (39.3)	285 (44.5)	
Student loans, n (%)			
Yes	179 (37.0)	238 (37.1)	.96
No	305 (63.0)	403 (62.9)	
Annual salary, n (%)			
≤$119 999	132 (27.3)	148 (23.1)	.40
$120 000‐$159 999	219 (45.3)	316 (49.3)	
≥$160 000	133 (27.5)	177 (27.6)	
State of employment, n (%)			
Top 10 states of COVID‐19 cases	206 (42.6)	266 (41.5)	.72
Type of health‐system, n (%)			
University	119 (24.6)	116 (18.1)	.004
Community, nonprofit	289 (59.7)	369 (57.6)	
Other (government, critical access, for‐profit, other)	76 (15.7)	156 (24.3)	
Number of beds in institution, n (%)			
0‐250	164 (33.9)	259 (40.4)	.029
251‐500	144 (29.8)	181 (28.2)	
501‐750	75 (15.5)	104 (16.2)	
>750	101 (20.9)	97 (15.2)	
Place of employment, n (%)			
Inpatient hospital	376 (77.7)	474 (73.9)	.39
Inpatient+ambulatory care clinics	41 (8.5)	73 (11.4)	
Ambulatory care clinics	33 (6.8)	48 (7.5)	
Other	34 (7.0)	46 (7.2)	
Current pharmacy position, n (%)			
Management	236 (48.8)	283 (44.1)	.12
Pharmacist (non‐management)	248 (51.2)	358 (55.9)	
Fulltime employment, n (%)	463 (95.7)	597 (93.1)	.072
Years of professional experience, n (%)			
0‐5	114 (23.6)	132 (20.6)	.53
6‐10	76 (15.7)	101 (15.8)	
11‐20	129 (26.7)	183 (28.5)	
20+	165 (34.1)	225 (35.1)	
Highest degree of training, n (%)			
B.S. or Pharm.D.	209 (43.2)	300 (46.8%)	.79
PGY1	159 (32.9)	210 (32.8)	
PGY2	102 (21.1)	116 (53.2)	
Other	14 (2.9)	15 (2.3)	
BPS certified, n (%)	247 (51.0)	355 (55.4)	.15
ACLS certified, n (%)	220 (45.5)	313 (48.8)	.26
Service areas for non‐managers, n (%)			
General medicine	45 (18.1)	79 (22.1)	.13
ICU	29 (11.7)	52 (14.5)	
Central pharmacy	28 (11.3)	35 (9.8)	
Emergency medicine	24 (9.7)	25 (7.0)	
Ambulatory care	20 (8.1)	30 (8.4)	
Medication safety	13 (5.2)	7 (2.0)	
Infectious disease	12 (4.8)	22 (6.1)	
Hematology/Oncology	12 (4.8)	19 (5.3)	
Pediatrics	10 (4.0)	11 (3.1)	
Other (transplant, surgery, investigational drugs, informatics, drug information, other)	55 (22.1)	78 (21.8)	

Abbreviations: BPS = B.S., Bachelor of Science; COVID‐19, coronavirus disease 2019; ICU, intensive care unit; PGY1, postgraduate year one; PGY2, postgraduate year two; Pharm.D., Doctor of pharmacy; SD, SD; ICU, intensive care unit.

As for the impact of the COVID‐19 pandemic on employment status and other related factors (see Table 2), all COVID‐19‐related factors significantly decreased in the 20‐month group compared with the early group. There was a reduction in the number of respondents with decreased salary or benefits (17.1% vs 8.4%), respondents who have lost childcare (33.8% vs 7.2%), and respondents who have been redeployed (8.1% vs 3.4%) or have been furloughed (5.8% vs 0.2%) in the 20‐month group compared with the early group. However, there was a significant increase in the number of respondents with increased work hours in the 20‐month group (57.6% vs 46.7%; *P* < .001).

**TABLE 2:** COVID‐19‐related factors

Factors	Early group (%)	20‐month group (%)	*P* value
Did your hours:			
Increase	226 (46.7)	369 (57.6)	<.001
Decrease	79 (16.3)	19 (3.0)	
Remain the same	179 (37)	253 (39.5)	
Furloughed			
Yes	28 (5.8)	1 (0.2)	<.001
Redeployed			
Yes	39 (8.1)	22 (3.4)	<.001
Decrease salary			
Yes	83 (17.1)	54 (8.4)	<.001
Have to work remotely			
Yes	187 (38.6)	59 (9.2)	<.001
Lose childcare			
Yes	73 (33.8)	29 (7.2)	<.001
Lose job			
Yes	6 (1.2)	6 (0.9)	.771
Significant other's job change/impacted			
Yes	129 (33.6)	87 (16.1)	<.001

Abbreviation: COVID‐19, coronavirus disease 2019.

There was a significant increase in the percent of respondents reporting that they have burnout based on the PWLS self‐rating in the 20‐month group compared with the early group (69% vs 47.7%, respectively; *P* < .001). There were more respondents in the early group who reported having a history of burnout compared with the 20‐month group (81% vs 69.9%, respectively; *P* = .006). Of respondents who reported burnout, significantly more respondents in the 20‐month group indicated that burnout was related to the pandemic compared with the early group (78.3% vs 51.2%, respectively; *P* < .001). As for duration of burnout when experienced, most respondents reported having burnout last for up to 12 months (81.3% in the early group vs 70.9% in the 20‐month group). However, there were significantly more respondents in the 20‐month group who reported having burnout last between 1 and 5 years (26.5% vs 17.7%; *P* < .001). Additionally, the perception of percent of pharmacists who are burned out in their institution increased by approximately 20% in the 20‐month group compared with the early group (60.9% vs 43.3%; *P* < .001), which is similar to their own self‐perceived burnout. The top five reasons and main drivers respondents believe pharmacists who are burned out were similar in both group and were due to: (1) workload, (2) efficiency and resources, (3) culture, (4) work–life integration, and (5) lack of rewards. Table 3 summarizes respondents' self‐ratings, perception, and description of burnout.

**TABLE 3:** Description of self‐reported burnout

Self‐reported burnout	Early group n (%)	20‐Month group n (%)	*P* value
Burnout self rating (n = 484)			
I have burnout	231 (47.7)	442 (69.0)	<.001
History of burnout (n = 253)			
Yes	205 (81.0)	137 (69.9)	.006
When you experienced burnout (or are currently), approximately how long did the symptoms last?			
Less than 3 months	148 (33.6)	100 (17.2)	<.001
3–12 months	202 (45.9)	312 (53.7)	
1–5 years	78 (17.7)	154 (26.5)	
>5 years	12 (2.7)	15 (2.6)	
If you are currently or have been recently burned out, is/was it related to the COVID‐19 pandemic?			
Yes	191 (51.2)	451 (78.3)	<.001
What do you believe are main drivers that have contributed most to job burnout in your institution?			
Workload	300	578	–
Efficiency and resources	257	371	
Culture	189	204	
Work–life integration	183	254	
Lack of rewards	133	172	
Control	128	111	
Meaning in work	118	109	
Flexibility	56	81	
Social support and community at work	55	65	
Other	33	59	
Perception of percent of pharmacists are burned out in their institution, mean ± SD	43.3 ± 23.1	60.9 ± 24.8	<.001

Abbreviations: COVID‐19, coronavirus disease 2019; SD, standard deviation.

Based on the ProQOL (see Table 4), almost all respondents were identified to have moderate–high likelihood of compassion satisfaction in the early and 20‐month groups (99.4% and 98.4%, respectively); however, there were more respondents in the 20‐month group with low likelihood of compassion satisfaction (1.6% vs 0.6%; *P* < .001). Additionally, more respondents in the 20‐month group were identified to have moderate–high likelihood of burnout (83.8% vs 65.3%, respectively; *P* < .001) and to have moderate–high likelihood of STS (63.2% vs 51.0%, respectively; *P* < .001). The median score of compassion satisfaction was 38 (17‐50) in the early group vs 36 (13‐50) in the 20‐month group (*P* < .001), burnout was 25 (10‐44) vs 28 (10‐44) (respectively; *P* < .001) and STS was 23 (11‐44) vs 25 (11‐47), all within the moderate likelihood range (23‐41).

**TABLE 4:** Description of ProQOL Scale categories

ProQOL categories	Compassion satisfaction, n (%)			Compassion fatigue, n (%)					
	Early group	20‐month group	*P* value	Burnout			Secondary traumatic stress		
				Early group	20‐month group	*P* value	Early group	20‐month group	*P* value
High (≥42)	117 (24.2)	102 (15.9)	.001	4 (0.8)	5 (0.8)	<.001	2 (0.4)	6 (0.5)	<.001
Moderate (23‐41)	364 (75.2)	529 (82.5)		312 (64.5)	532 (83.0)		247 (51.0)	405 (63.2)	
Low (≤22)	3 (0.6)	10 (1.6)		168 (34.7)	104 (16.2)		235 (48.6)	230 (35.9)	
Median score (range)	38 (17–50)	36 (13‐50)	<0.001	25 (10‐44)	28 (10‐44)	<0.001	23 (11‐44)	25 (11‐47)	<.001

Abbreviation: ProQOL, Professional Quality of Life.

## 4 DISCUSSION

Several studies have shown that pharmacists are burned out during the COVID‐19 pandemic.^5^, ^6^, ^7^ A recent study (n = 439) showed that pharmacists reported increased feelings of physical exhaustion at work (45%) and of emotional exhaustion at work (53%) during the pandemic.^7^ Additionally, approximately 40% of pharmacists reported feeling more anxiety and 25% feeling more depression or sadness. Another recent study in pharmacists (n = 647) showed that the mean Maslach Burnout Inventory (MBI) scores for emotional exhaustion (28.5) and depersonalization range (7.98) were higher than reported pre‐COVID‐19 scores, which may indicate increased burnout.^6^ Pharmacists reported that working overtime, medication supply and patient incivility were factors that affected work. This has been shown in several recent studies in nurses, physicians, and other health care professionals throughout the world.^11^, ^12^, ^13^, ^14^, ^15^, ^16^, ^17^, ^18^ A recent study assessed the psychological response in 467 nurses early in the COVID‐19 pandemic and showed that 54.6% of nurses reported traumatic stress, 54.6% reported depressive symptoms, 32.4% reported insomnia, and 37.3% reported anxiety.^17^ Another study in 605 health care workers (physicians, nurse practitioners, nurses, physician assistants, patient care technicians, respiratory therapists, social workers, mental health therapists, and case managers) early in the pandemic showed that 14.2% reported depressive symptoms, 43.2% reported mild or high anxiety, 22.3% reported posttraumatic stress disorder (PTSD) symptoms, 46% reported emotional exhaustion, 21.6% reported depersonalization, and 23.1% reported lower resilience.^18^ Health care workers who cared for COVID‐19 infected patients in‐person were more likely to experience worse depression, anxiety, possible PTSD, and higher burnout.

Our study is the first, to our knowledge, that assessed the changing pattern and impact of burnout and STS in health‐system pharmacists throughout the COVID‐19 pandemic. The results showed that health‐system pharmacists have significantly higher rates of burnout and STS 20 months into the pandemic compared with rates reported earlier in the pandemic. Self‐perceived burnout increased by 21% from early in the pandemic (April to May 2020) to 20 months into the pandemic (October to December 2021) (*P* < .001). Additionally, almost 80% of respondents in the 20‐month group reported that burnout was related to the COVID‐19 pandemic, which increased from 51.2% in the early group (*P* < .001). Unfortunately, there was a significant increase in respondents who reported burnout duration of 1‐5 years in the 20‐month group compared with the early group (26.5% vs 17.7%; *P* < .001). Additionally, there was a higher number of pharmacists in the 20‐month group with a moderate–high likelihood of burnout (83.8% vs 65.3%; *P* < .001) and a higher number of pharmacists in the 20‐month group with moderate–high likelihood of STS (63.2% vs 51.0%; *P* < .001), which is a concern. Inversely, almost all pharmacists had moderate–high likelihood of compassion satisfaction; however, there were more pharmacists 20‐months into the pandemic with low likelihood of compassion satisfaction. These results indicate that in the setting of high rates of burnout and STS most likely due to the COVID‐19 pandemic, compassion satisfaction may be impacted. Additionally, regardless of the improvement in COVID‐19‐related factors, our results showed burnout and STS worsening 20‐months into the pandemic. Based on these results, further studies are needed in assessing effective interventions to address the impact of the COVID‐19 pandemic on burnout, STS, and compassion satisfaction. There are limitations with our study which included that the length of the survey perhaps caused fewer responses; but almost 80% (1126/1421) of respondents completed the survey. Although an accurate response rate could not be calculated, the response rate would most likely be low which increases risk of selection bias and would limit generalizability of the study results. Additionally, about 50% of the respondents were in management positions which would also limit generalizability of the results. However, the strengths of our study included having a comparator group (early in the pandemic vs later in the pandemic) to assess the changing patterns and effects of burnout and STS throughout the pandemic. To our knowledge, this is the first study that assessed the changing patterns of the prevalence of burnout and STS in health care professionals, especially health‐system pharmacists, throughout the COVID‐19 pandemic.

Overall, our study is unique compared with previous studies in health‐system pharmacists because we identified the changing patterns and impact of burnout, STS, and compassion satisfaction throughout the COVID‐19 pandemic. Additionally, we reported that the overall prevalence of burnout in health‐system pharmacists is over 80% of respondents and for STS is over 60% of respondents 20 months into the pandemic. These rates are significantly higher compared with rates early in the pandemic. Unfortunately, we know that the development of burnout and STS may lead to work‐related consequences such as decreased productivity, quality of patient care and patient satisfaction, increased employee turnover, and more concerning, increased risk of medical errors, substance abuse, depression and suicide, and disrupted relationships.^3^ Therefore, it is crucial to develop and assess effective interventions to address burnout and STS in these health‐system pharmacists.

## 5 CONCLUSION

Twenty months into the COVID‐19 pandemic, almost 70% of health‐system pharmacist respondents identified as being burned out, and this is reflected by the high percentage of pharmacists with moderate–high likelihood of burnout based on the ProQOL. Additionally, a high percentage of health‐system pharmacists had moderate–high likelihood of STS, but compassion satisfaction scores were lower later in the pandemic. This shows that we are seeing increased rates of burnout and STS, and compassion satisfaction may be impacted the further we are into the COVID‐19 pandemic. Further studies are critical to develop and assess effective interventions to address the effects of the COVID‐19 pandemic and the well‐being of health‐system pharmacists.

## CONFLICT OF INTEREST

The authors declare no conflicts of interest.
